# An archive of childhoods outside the norm: a perspective on Suzanne Osten’s theatre for young audiences

**DOI:** 10.3389/fsoc.2025.1610174

**Published:** 2025-06-19

**Authors:** Rebecca Brinch

**Affiliations:** Department of Culture and Aesthetics, Stockholm University, Stockholm, Sweden

**Keywords:** Suzanne Osten, theatre for young audiences, trauma, sideways growth, childhood and gendered mental health issues

## Abstract

This article discusses how childhood as well as gendered mental health issues are represented in Swedish director Suzanne Osten’s theatre for young audiences. The article illustrates examples from two of Osten’s theatre productions: *The Girl, The Mother, and the Garbage* (1998, for ages seven and up) and *The Baa-Lambs Holiday* (2014, for ages 15 and up). The article argues that Osten’s approach and dramaturgical strategies in her theatre productions are crucial for addressing topics many consider taboo for young audiences. Osten’s theatre performances can be regarded as a collective and ongoing contribution to public media debates surrounding children’s culture and the child’s place in society at large. It contends that her theatre for young audiences can be characterized as an “archive of feelings” of childhoods that are otherwise seldom highlighted in public.

## Introduction

Suzanne Osten, born in 1944 in Stockholm, passed away in October 2024 at the age of 80, at the height of a successful career in theatre and film, with several planned and ongoing projects. Osten’s significance for Swedish and international theatre can not be overestimated. She established Unga Klara (Young Klara) in 1975, a theatre group that since 2018 serves as Sweden’s national stage for performing theatre for children and young audiences. Suzanne Osten created feminist and politically engaged theatre (Svens, 2002; Rosenberg, 2004, 2009; Johansson, 2006). In the 1970s and onward she collaborated with Margareta Garpe on the theatre performances *Girltalk* (*Tjejsnack,* 1971), *The Love Performance* (*Kärleksföreställningen,* 1973), *Gosh, Girls: Liberation is Nigh* (*Jösses flickor, befrielsen är nära!,* 1974), *The Factory Girls: The Power and the Glory* (*Fabriksflickorna: Makten och härligheten,* 1980) and *Gosh, Girls: The return* (*Jösses flickor: Återkomsten,* 2006). All these performances aimed to raise feminist awareness by questioning patriarchal structures and highlighting marginalized voices (Johansson, 2006; Rosenberg, 2009). Osten declares that: “I have dedicated myself to listening to and portraying the powerless: children, women, refugees, and the destitute. It is the task of the theatre to find voices that are not heard. We do not live in a democratic storytelling tradition where everyone has a say” (Göteborgs Stadsmuseum, 2016). This involves portraying childhood from a child’s perspective, exploring how, within a generational system where adulthood is considered the norm, childhood is often viewed as deviant (Alanen, 2009). In my doctoral dissertation, I examined notions of the child, artistic expressions, and strategies employed in Osten’s theatrical productions for young audiences, covering almost 30 years of her career (Brinch, 2018). The dissertation shows that key themes in Osten’s theatre for young audiences include the depiction of trauma, the concept of age and generation, and the role of art as imperative to children’s well-being. In Osten’s work, strong emotions and existential questions are portrayed from a child’s perspective, placing the child’s experiences at the forefront. Osten’s theatre examines age and generational dynamics in various ways, highlighting different power structures associated with age. In her work, children’s everyday lives and conditions are portrayed, illustrating how they frequently face challenging circumstances and feel powerless when confronted with adults’ decisions. Regarding art as imperative for children’s well-being, Osten depicts emotional isolation as the worst thing a child can be subjected to, while art is portrayed as a possible survival strategy. This connects to psychoanalyst D. W. Winnicott’s theories about transitional space as an area for play, creativity and cultural experiences where children (and adults) can process their experiences (Winnicott, 1995).

Even though Osten’s artistry is recognized internationally, little research has been conducted on her outside of Sweden. Three doctoral dissertations explore her work: one examines her directing from a feminist perspective (Svens, 2002); another investigates her collaboration with Margareta Garpe in the 1970s at The Stockholm City Theatre (Johansson, 2006); and finally, my own centers on artistic expression and aesthetic strategies (Brinch, 2018). Several words and phrases frequently appear in the descriptions of Suzanne Osten’s theatrical work: taboo subjects, feminism, radicalism, political, modernist, theatre as art, a child’s perspective, and power (Rosenberg, 2009, 2012; Helander and Zern, 2010; Helander, 2018, 2024; Helander and Osten, 2010; Elnan, 2009; Van de Water, 2012; Švachová, 2016). Additionally, many more expressions could be included, as Osten consistently reflects on her work in different articles and books, both on her own and in edited volumes (Osten, 2006, 2013, 2021; Rosenberg, 2004; Helander and Osten, 2007; Sparby, 1986; von Zweigberg, 1990). When I met Osten during a conversation in the spring of 2014 and inquired about important themes in her artistry, she responded quickly and without hesitation: “Children and power.” And added: “As political as possible.”^1^

## Suzanne Osten’s theatre for young audiences

This article aims to discuss the representation of childhood and gendered mental health issues in Suzanne Osten’s theatre for young audiences. The article utilizes theories of sideways growth and trauma to analyze Osten’s artistic choices, exploring how these decisions influence the portrayal of childhood and gendered mental health issues in her work. Additionally, the article illuminates Osten’s methods and the dramaturgical strategies she uses, emphasizing their importance in addressing topics often regarded as sensitive or inappropriate for children. The article argues that Osten’s performances for young audiences can serve as an “archive of feelings,” reflecting childhoods frequently overlooked in public media debates. The article is an extension of my doctoral dissertation (Brinch, 2018). While it summarizes key findings of my previous analyses, its novelty lies in offering a new perspective on Osten’s theatre for young audiences by focusing specifically on childhood and gendered mental health issues. This adds a new dimension to research findings on Osten’s body of work.

To begin with, it is important to recognize that professional theatre for children is created within an adult-centric context, with adults determining what constitutes “good” or “bad” theatre for children (Helander, 1999). These opinions shift over time, indicating that theatre for children goes beyond what is merely presented on stage. It is deeply intertwined with the surrounding society, cultural norms, and ideas about childhood. Studying theatre for children as an artistic expression also involves analyzing how we view children and different ideas about age, growth, and what can be considered suitable for children to see on the theatre stage (or not). The perception of children as vulnerable and powerless, and as a group in society that needs protection (Lee, 2001), also impacts children’s theatre. Rather than focusing on the real challenges that children face in the present, theatre for children can prioritize entertainment or mirror adults’ nostalgic views of childhood. This is, however, not the case in Suzanne Osten’s theatre for young audiences. As previously shown, Osten consistently staged themes that can be considered taboo for children (Brinch, 2018; Helander, 1999; Helander, 2024). This includes highlighting gendered mental health issues by staging productions emphasizing the societal pressures that women and girls face. Notable productions include: *Medea’s Children* (*Medeas barn,* 1975), which tells the story of little Medea and little Jason who, during their parent’s divorce, play games about self-harm; *A Clean Girl* (*En ren flicka,* 1983), exploring anorexia and self-starvation; *The Girl, The Mother, and the Garbage* (*Flickan, mamman och soporna,* 1998), about a girl growing up with a mother who has a mental illness; *Borderline* (*Gränsen,* 2000), centered on two young girls who commit suicide; *Horse, Horse Horse - about Leonora Carrington* (*Häst, Häst, Häst – om Leonora Carrington,* 2013); and *The Baa-Lambs Holiday* (*Lammungarnas Fest,* 2014), also focusing on the artist and surrealist Leonora Carrington, who struggled with mental illness throughout her life; and *Anatomy of a Suicide* (*Ett självmords anatomi,* 2019) which examines the relationship between mothers and daughters and raises questions about whether suicidal tendencies can be inherited.^2^

The article analyzes examples from two of Osten’s theatre productions: *The Girl, The Mother, and the Garbage* (for ages seven and up), and *The Baa-Lambs Holiday* (for ages 15 and up); additionally, a third production, *Medea’s Children*, is mentioned in order to exemplify societal debates generated by Osten’s work.^3^ *The Girl, The Mother, and the Garbage* is based on Osten’s upbringing with a single mother who suffered from schizophrenia. The performance was adapted by Erik Uddenberg from a book by Osten.^4^ The production was performed at Unga Klara in 1998 and 1999 for an audience consisting of school children. In the evenings and on weekends, public performances were held for mixed-age audiences. The production went on tour in Sweden and also toured Germany, Canada, South Africa, and the USA, where it was presented in English. *The Baa-Lambs Holiday* was originally written by surrealist painter and writer Leonora Carrington in the 1940s, and adapted by Erik Uddenberg and Suzanne Osten.^5^ The production explores Leonora Carrington’s life as an artist and woman within a patriarchal society, showcasing how expectations of women and their limited roles contributed to Leonora’s illness and breakdown in different ways. The performance was staged at Unga Klara in 2014, and was Osten’s final production as the artistic director of Unga Klara. *The Baa-Lambs Holiday* was performed for young people in middle or high school. Several public performances were also given, with a mixed age audience.

## The wish to communicate and to work with a child’s perspective

One characteristic of Osten’s artistry is a focus on the audience and communication with the spectators (see also Rosenberg, 2004). This interest started early and can be traced back to the 1960s when Osten studied art history at Lund University and founded the theatre group Fickteatern (“The Pocket Theatre”). Fickteatern was one of Sweden’s first independent theatre groups, and it was through this fringe group that Osten started making work for young audiences. According to Osten, she discovered that the young audience was much more emotionally engaged when they saw theatre that, in one way or another, related directly to their own life-world context. When Osten started Unga Klara in 1975, she also started working with what would later be one of the trademarks of this theatre company: reference groups and test audiences. A test audience is used before a production’s official premiere, allowing adjustments to be made to ensure that the play is suitable for the intended age group, while reference groups follow the rehearsal process from early on (Lorentzon et al., n.d.). The primary purpose of working with reference groups is to test different ideas and discuss questions together, using the children as expert consultants, to confirm that the production is heading in the right direction and effectively communicates with its young audience. Before each production, extensive research is also conducted related to the theme being portrayed. Osten particularly highlighted the importance of reference group work. Her conviction was that to portray children’s worlds from a child’s perspective, a dialogue with the target group is required, and this work should start as soon as the work with a theatre production starts. Comprehensive research is carried out, often in collaboration with the young audience. The rehearsal processes included the entire theatre ensemble, actors, dramaturgs, scenographers, and playwrights, and spanned over a period that lasted longer than the average rehearsal time in theatres. According to Osten, working with a child’s perspective requires that every action is examined from the child’s point of view, hence the importance of working with reference groups. Osten’s work with a child’s perspective could also be defined as “the willingness of the adult world to highlight and make visible children’s conditions” (Helander, 2024, p. 160), and, as Helander notes, Osten did this a long time before the concept was formulated by the United Nations Convention on the Rights of the Child (Helander, 2024, p. 160).

## Performing childhood and gendered mental health issues

In *The Girl, The Mother, and the Garbage*, Osten depicts experiences from her childhood and her upbringing with a mother who has mental health challenges.^6^ In the play, we are introduced to Ti, a seven-year-old girl living with her mother, who is facing challenges with schizophrenia. In the play, the mother’s illness is portrayed by two demons, Mister Polter and Mister Geist. Throughout the performance, we observe the demons tormenting Ti’s mother, which leads her, due to her illness, to collect garbage, and prevent Ti from attending school, confining both of them to their apartment. The play concludes ambiguously; we see Ti and her mother facing eviction and the child being rescued from the cramped apartment, yet we do not learn what happens to Ti and her mother afterward.

In the performance, the mother’s mental illness is depicted from a child’s perspective. However, the portrayal of Ti’s mother’s struggles with mental illness is not shown as something private, hidden away within the apartment’s four walls. Instead, the experiences are framed within a broader context. Ti reflects on how her mother’s sickness, saying: “The kind who walk around town and collect a lot of trash, with overflowing plastic bags and a cart with wheels on it. That’s how my mother is now. Sometimes, at night, I hear her crying.”^7^ Ti understands that her mother is unwell and wants to protect her, but at the same time, she only wants to go to school. Ti’s mother is fearful of other people, believing they will harm her, and advises Ti not to stand out: “Come here. Remember. We have to act normal. Look normal. Not deviate. Not be heard. It’s important to be whole and clean.” In one instance, Ti’s teacher inquires about her well-being at home and suspects things are not good. However, the teacher does not seek further help until Ti’s mother’s condition worsens, leaving Ti unable to go to school.^8^ There are several scenes like this in the theatre performance, where it becomes evident that others can sense that something is not right, yet they choose not to ask questions before things have worsened significantly. In the end, the mother refuses to go out and locks herself in the apartment together with Ti. This leaves Ti feeling utterly abandoned both by society and her mother: “It’s as if the school does not exist anymore. Maybe there’s nothing left except for what I can see right now. The only thing that exists is this room.” Throughout the performance, it is clear that neither Ti nor her mother is receiving the support they require. Consequently, the home environment depicted in the theatre performance reflects a broader political and social context, beyond merely portraying a mother who neglects her child. It also addresses a society that overlooks individuals struggling with mental health issues, including children who grow up in the shadow of their parents’ illness. While Osten was working on the production, she was in contact with the organization Källan, which supports children of mentally ill parents. The organization was established as a pilot project in response to the inadequate support for children growing up with mentally ill parents within the healthcare system. During the development of the theatre production, children involved in Källan’s activities were also included in reference groups.^9^

It is key that the character portrayal in *The Girl, The Mother, and The Garbage* is of a mother rather than a father. Typically, the mother’s role is expected to provide security and care; however, Ti’s mother struggles to fulfill this expectation due to mental illness. The fact that Ti lives alone with a mother, who is unable to provide the care and emotional security traditionally associated with motherhood, underscores the child’s vulnerability. Equally important is the play’s illustration of the mother’s struggle and the challenges she faces, which ultimately contribute to her breakdown and mental illness. This reveals how the mother’s health challenges are intertwined with societal expectations of women and the traditional roles assigned to them, particularly as mothers.^10^ In a dissertation on the impact of childhood conditions for children with a parent suffering from mental illness, scholar and social worker Annemi Skjerfving highlights the taboos surrounding discussions of this issue with children (Skjerfving, 2015). She points out that children are more affected when it is the mother who has a mental illness, likely due to expectations and parental roles for women and men in Sweden.^11^ Skjerfving emphasizes that understanding the parents’ issues can enhance the child’s ability to cope and reduce feelings of guilt and responsibility for the parents’ condition. *The Girl, The Mother, and the Garbage* accentuates a society that fails both the mother and the child. The production not only depicts a childhood outside the norm, providing children in the audience who have similar experiences the opportunity to voice their situations; additionally, it highlights societal expectations surrounding women and motherhood. When the play was staged in 1998, a public investigation by the state into the needs of children was conducted, and discussions about democracy focusing on children’s right to culture were held at a major international congress in Stockholm.^12^ *The Girl, The Mother, and the Garbage* can be seen as a contribution to an ongoing debate about the welfare state’s responsibility toward children, where Osten, through theatre, emphasizes the interconnectedness and vulnerability of children and women’s mental health.^13^

Questions of childhood and gender mental health issues are framed somewhat differently in *The Baa-Lambs Holiday*. In this play, we meet Teodora, a teenager who rebels against the bourgeoisie and the nuclear family, refusing to conform to the contemporary expectations of young women. The play is closely connected to the real Leonora Carrington’s (1917–2011) life and upbringing in England. She grew up in an aristocratic family from which she rebelled and eventually escaped. She was an artist and active participant in the surrealist movement during a time when the art world was predominantly male.

In Osten’s version of *The Baa-Lambs Holiday*, we first encounter Leonora herself on stage, although the plot centers around Teodora, Leonora’s alter ego, who reflects on her own upbringing. The performance is filled with references to art and creativity. The scenography features picture frames that actors either inhabit or move through as they transition from one scene to another. Leonora occasionally walks around the stage with a paint palette and brushes, while Teodora is constantly driven by a desire to create. The character Leonora serves as a link between Teodora and the audience, offering commentary on the events that unfold, which parallels the experiences of the character Teodora. *The Baa-Lambs Holiday* is a surrealist drama filled with animals, anger, blood, and creativity, where Teodora resists the pressures of the adult world to “grow up.” Instead, Teodora grows sideways according to her terms and desires, shaping a life different from what is expected. The concept of growing sideways is borrowed from Bond Stockton (2009) and her discussion of the queer child. Stockton suggests that the queer child’s growth happens sideways, with movements and directions beyond just upward toward marriage, reproduction, and profitability.

By allowing Teodora to make different life choices than those normatively expected, thereby illustrating a sideways, not upward, growth, Osten exposes societal norms regarding gender and mental health issues, and how these constraints affect the well-being of both girls and women. In *The Baa-Lambs Holiday*, Teodora resists her mother-in-law’s attempts to label her as mentally unstable simply because she refuses to submit to the expectations of her husband. Teodora’s mother-in-law, Mrs. Kött, repeatedly comments on Teodora’s behavior, telling her that she needs to stop being so childish and grow up: “Is that how one behaves? A well-bred young girl! Honestly, Teodora.”^14^ When Teodora continues to refuse to act like the obedient wife her mother-in-law expects, Mrs. Kött labels her mentally unstable; in the background, there is the added threat of Filip’s former wife, who has disappeared without anyone understanding why or how. Mrs. Kött tells Filip: “If I were you, I would give her [Teodora] a proper overhaul. I do not approve of escapades like tonight at all.” Despite these threats, Teodora continues to assert her independence throughout the performance. In *The Baa-Lambs Holiday*, Osten invites the audience to take a critical stance toward simplistic and dangerous categorizations and to recalibrate what is viewed as “normal” or not. Teodora repeatedly refuses to conform to the world’s expectations of how she should behave. She prefers spending time with animals rather than with her husband or mother-in-law, and ultimately, she runs away with the werewolf Roland. In the final scene, she leaves Roland and chooses not to take the expected step into normative adulthood; instead, she opts to forge her own path. She starts singing and playing an electric guitar, inviting the audience to join in or to create on their own. In this way, Teodora prioritizes her creativity and independence over her marriage and the life she was supposed to live, growing sideways into her artistry and who she wants to be. In many ways, the theater production can be viewed as the character Leonora (who symbolizes the real Leonora Carrington) being allowed, through Teodora, to invent new versions of herself and recreate her history by growing sideways. In this manner, creativity, integrity, and autonomy are central to the production, which becomes a narrative about liberation and survival through art. In an interview about the play, Osten said: “I think we mislead young people when we say they have to be useful all the time. We have a fairly aggressive education policy, where we remove all aesthetic elements. It is often a survival skill in adolescence to be able to express yourself artistically.”^15^ This can also be seen in light of gendered mental health issues, where *The Baa-Lambs Holiday* encourages the audience not only to see how restrictive gender norms and gender inequalities can have serious health implications on women’s lives, but also to reimagine this. Thus, by making various life experiences visible, time is presented innovatively in *The Baa-Lambs Holiday*, reflecting a type of queer temporality that challenges conventional perceptions of time and raises questions about how life can and should be lived. New childhoods emerge in Osten’s productions. Additionally, the play illustrates how sideways growth can happen at any age, allowing Leonora to reimagine and relive her experiences through Teodora. This might not only open up possibilities for the children in the audience but also for adults, where it becomes feasible to grow sideways, even as an adult, by one’s desires and will.

To conclude, both performances engage with questions surrounding childhood and gendered mental health issues in distinct ways. The gendering of mental health is explored through the portrayal of a daughter and a mother (*The Girl, The Mother, and The Garbage*) and a teenage girl (*The Baa-Lambs Holiday*), and by illuminating different societal expectations on girls and women and highlighting how the pressure and expectations placed on these groups are connected to mental illness in various ways. In *The Girl, The Mother and The Garbage,* we meet Ti, who navigates contemporary life, and in *The Baa-Lambs Holiday,* we encounter Teorora in 1930s England. Each play presents various ideas about childhood and gendered mental health issues from different historical times. Yet, they also present solutions: Teodora finds freedom by growing sideways instead of up, and Ti is acknowledged and validated in her trauma. In this way, both plays suggest alternatives to stereotypical representations of teenage girls, daughters, and mothers, who, in different ways, struggle with issues of mental illness. Additionally, childhood and gendered mental illness are connected in the plays by foregrounding the perspectives of the daughter and the teenage girl, allowing the audience to follow the narrative from the child’s point of view. As such, both plays can help spark discussions about childhood and gendered mental health issues, addressing expectations on how girls and young women’s lives should be lived, and the role and conditions of children growing up.

## Staging thematics that can be considered taboo for young audiences

How does one stage thematics that can be considered sensitive or inappropriate for young audiences? To begin with, Osten’s theatre for young audiences has sparked considerable debate regarding the themes portrayed on stage. *The Girl, The Mother, and the Garbage* were criticized for addressing themes deemed too challenging for children, and the film, based on the production that premiered in 2016, was initially banned for children. Osten appealed this decision, and, as a result, the film was approved for children from 11 years old (Helander, 2024). Similar debates have arisen around several of Osten’s theatre productions aimed at children. One example is the debate surrounding *Medea’s Children* (*Medeas barn,* 1975) that depicts divorce from a child’s perspective. In the play, the siblings Little Jason and Little Medea are forced to act like adults while their parents, Jason and Medea, are caught in a crisis that leaves the children to fend for themselves. Their nanny, Anna, aids them in the ordeal, and ultimately, the adults confront their new reality and attempt to resolve their crisis. Some critics argued that the play portrayed too many intense emotions for the young audience (7 years and up). Unga Klara received letters from teachers and parents who felt that the themes of the play were not suitable for children. Despite this criticism, the play also garnered a great deal of praise (Helander, 1999). Despite the controversy, Osten’s plays continue to be performed, and many of her advocates emphasize the importance of showcasing the darker sides of childhood alongside the brighter ones. Throughout her career, Osten has consistently shown a rare ability to address topics few others would approach. It is also clear that the debate often overlooks that children and adults do not always share the same perception of what can be frightening or challenging (see, for example, Helander, 2011; Lidén, 2022). These discussions often center on whether themes are age-appropriate, while the form and aesthetics of the performances are overlooked. In my research, I argue that Osten uses specific dramaturgical strategies to adapt what is portrayed for different age groups, consistently placing the child character in a context that cultivates feelings of reassurance for the young audience (Brinch, 2018). Importantly, these strategies often employ retrospective storytelling. This technique allows Osten to create a necessary distance from the emotionally intense themes being presented. In *The Girl, The Mother, and the Garbage*, we encounter Ti as an adult before the performance starts. From the outset, this reassures the audience that Ti and her mother will survive. This establishes a certain distance before we enter the cramped apartment where Ti is confined with her mother. Another instance of retrospective storytelling in the performance is how Ti functions not only as the daughter but also as the narrator. At times, Ti steps away from the linear narrative of the drama and explains to the audience, as grown-up Ti, more neutrally what is happening to her and her mother. This means the narration alternates between a first-person perspective and a more distant, retrospective view. In this way, much-needed distance is created for the audience, allowing for reflection on what is depicted. It also reassures that Ti will survive since she exists both as a child and an adult on stage in a parallel narrative universe.

In *The Baa-Lambs Holiday*, we meet Leonora in the foyer before the performance starts, where she informs us that the play we are about to see is based on her life. An experience of retroactive storytelling is created as she takes us by the hand and guides us through the various stages of her childhood story as acted out on stage by her younger alter-ego Teodora. Other dramaturgical strategies that Osten employs in her theatre for young audiences include childhood play as a form and a medium of communication. In Osten’s work, there are always elements of play that can be understood as an effort to establish a mode of communication that resonates with the young audience. Play as a form also allows for using dramaturgical models beyond the traditional. Utilizing play as a dramaturgical strategy permits breaks; time can be portrayed as non-linear, fragmented narratives can be employed, and open, fluid endings can be utilized, just like in children’s play (Gjervan, 2013). An example of elements of play dramaturgy in Osten’s productions can be found in *The Girl, The Mother, and the Garbage*, where the depiction of the mother’s schizophrenia is illustrated through two demons, Mister Polter and Mister Geist (with a clear nod to film as a medium). This strategy not only emphasizes the theatrical and playful elements of the production but also contributes to placing the illness outside the mother. For the young audience, the blame is shifted away from the mother, making it clear that her options are limited. The illness controls her, not the other way around. Play is also utilized as a dramaturgical strategy in *The Baa-Lambs Holiday*, where time is depicted as fluid and non-linear. We met Teodora both as a woman and a child, always playing, refusing to let anyone tell her how to behave and what to be, and growing sideways.

## Osten’s theatre for young audiences as an “archive of feelings”

Children who experience childhood outside normative expectations often lack representation and reflection on stage (Brinch, 2018). Suzanne Osten’s theatre for young audiences is an exception to this, and now, as we can view Osten’s work in light of her death, her theatre for young audiences can be characterized as an “archive of feelings” of childhoods that otherwise seldom are highlighted in public discussions. The term should be understood following Ann Cvetkovich’s use of the concept (2003). Cvetkovich analyzes lesbian sites of trauma and explores how these sites “give rise to different ways of thinking about trauma and in particular to a sense of trauma as connected to the textures of everyday experience” (Cvetkovich, 2003, p. 5). She defines trauma broadly and connects it to social and political structures, arguing that traditional trauma studies have overlooked the experiences of women and queers (and, I argue, children). Trauma is not limited to catastrophic events; it can also involve navigating daily encounters with, for example, racism, sexism, and ageism. According to Osten, childhood itself can be understood in relation to its lack of autonomy and power, where children face prejudices and discrimination as a social group compared to adults.^16^ By linking trauma and its feelings to the public sphere, Cvetkovich challenges the notion of trauma as solely an individual symptom. She illustrates how trauma has cultural and societal connotations, where certain feelings are validated, while others are pushed into the private sphere, associated with the individual instead of being recognized as part of structural oppression. Cvetkovich underscores the importance of recognition, as trauma can be viewed as a form of emotional memory. She builds on the idea that it is difficult to develop a sense of collective identity without an archival memory to support it and argues that certain groups and traumas in society are considered worthy of being included in national archival memory, while others are overlooked. According to Cvetkovich, theatre can provide a platform for public counterculture, revealing marginalized experiences and granting individuals the recognition that traditional trauma research identifies as essential for personal healing. Cvetkovich highlights the importance of recognition, as trauma can be understood as a form of emotional memory.

Following Cvetkovich’s perspective on trauma and public culture (2003), it becomes possible to describe Osten’s theatre performances for young audiences as an “archive of feelings,” drawing attention to the experiences of children (and women) while simultaneously serving as a potential means of processing trauma.^17^ Perhaps this is also why Suzanne Osten’s work resonates so profoundly on an emotional level. Creating an “archive of feelings,” Osten contributes to the acknowledgment of individual experiences, as well as developing a shared history and collective identity. Osten’s theatre for young audiences has politically radical potential because it brings what is considered private into the public arena. At the time Osten did this, it was pioneering. In today’s era of social media, reality TV, and documentary theatre, where intimate practices are being performed, the situation has shifted somewhat to blur the line between public and private. However, it is interesting to note that ideas about childhood seem to be difficult to change. For example, the debate surrounding *Medea’s children* that first emerged when the theatre production premiered in 1975 resurfaced nearly 40 years later in 2015 when the production was re-staged in Örebro.^18^ Another case, already discussed, is the film *The Girl, The Mother and the Demons*, which was initially banned for children but later allowed for those aged 11 and older. In Osten’s theatrical oeuvre, childhood is portrayed as a complex phenomenon. As such, her performances continue to provoke reactions even today, disrupting the distinctions between child and adult and private and public, offering new insights into how we can comprehend childhood.

## Data Availability

The dataset used for this publication consists of recordings of theater performances and an interview. The author holds the right to the interview, which can be made available upon request. The recorded theater performances and accompanying manuscripts can be requested from Unga Klara, https://www.ungaklara.se/, and Kulturhuset Stockholms Stadsteater, https://kulturhusetstadsteatern.se/.

## Footnotes

^1^Interview with Suzanne Osten, April 29, 2014.

^2^Some performances mentioned are original works by Osten, alone, or in collaboration, while others are adaptations of existing works. Titles in my translation.

^3^It should be noted that the two performances discussed in this article are also analyzed in my thesis. I expand on these analyses in the article, providing new insights by focusing solely on childhood and gendered mental health issues. Furthermore, the focus is exclusively on Osten’s work with the performances. However, Osten would have been the first to acknowledge that theatre emerges as a result of teamwork and that the two performances were, by definition, a joint effort by a team of members.

^4^The performance is based on a children’s book by Osten and Högberg (1998). The drama text of the play *The Girl, The Mother and The Garbage* can be found in Osten (2009).

^5^The work with the production *The Baa-Lambs Holiday* is described in a book by Osten et al. (2014).

^6^In 2016, the story was adapted into a movie directed by Suzanne Osten titled *The Girl, the Mother and The Demons* (*Flickan, mamman och demonerna*).

^7^All lines cited from *The Girl, The Mother, and The Garbage* are from the drama text, in my translation. The text can be found in Osten (2009).

^8^In the end, Ti’s teacher comes to aid when she raises the alarm that Ti is no longer coming to school.

^9^Osten, *Dagens Nyheter*, 1998.

^10^Osten also made the film *Mother* (*Mamma,* 1982), which is based on her mother Gerd Osten’s diaries and depicts her life and circumstances in 1940s Sweden. The film illustrates how a patriarchal society makes it challenging for women to combine parenthood with professional work. See Svensk Filmdatabas (2025).

^11^The thesis is based on interviews with children who have a parent with mental illness. The interviews were conducted in Sweden in the late 1990s.

^12^The Child and Adolescent Psychiatry Inquiry was commissioned by the state, SOU (1997): 8. The final report was submitted in 1998, SOU (1998):31.

^13^Several pre- and post-talks were given as part of the production. The production also included educational material aimed at teachers and school students, which could be used before and after attending the theatre performance. This material included interviews with children from Källan and information on what adults can do to help children living with a parent who has a mental illness. *The Girl, The Mother, and The Garbage* was also part of The Mental Health Campaign, a collaboration between The Equality Ombudsman (in the 90s called Handikappombudsmannen), The National Association for Social and Mental Health (Riksförbundet för social och mental hälsa), and The Swedish Schizophrenia Association (Schizofreniförbundet). The campaign aimed to raise awareness about mental illness. One of the main elements of the campaign was culture, with *The Girl, the Mother, and the Garbage* included as a feature.

^14^All lines cited from *The Baa-Lambs Holiday* are from the manuscript of the play, in my translation. In this article, I have chosen not to include stage directions in the lines. The manuscript is titled “Leonora Carrington. Lammungarnas fest. Översättning från engelskan Staffan Holmgren. Bearbetning Suzanne Osten och Erik Uddenberg (under bearbetning) 2014-01-20.” The author thanks Unga Klara for access to the manuscript.

^15^*Sydsvenskan,* 2013. During this time, aesthetic subjects had become non-compulsory for high school students. As a result, many schools saw a complete disappearance of these subjects, since students increasingly chose not to enroll in them. This trend can be attributed, among other reasons, to the fact that other subjects offer more merit points. For more information, Svenska Dagbladet (2018).

^16^See, for example, Young-Bruehl, 2013.

^17^The term” archive of feelings” originates from Cvetkovich (2003).

^18^The production in question was directed by Michael Cocke at Örebro Teater. Several performances were canceled after parents had raised concerns about the theme of the play, and Osten wrote a debate article about this as a reply: Osten, *Dagens Nyheter*, 2015.

## References

[ref1] Sydsvenskan “Att leva är att ta smällar”. (2013). Available online at:https://www.sydsvenskan.se/artikel/att-leva-ar-att-ta-smallar/ [Accessed 17 May 2025].

[ref2] Alanen L. (2009). “Generational order” in The Palgrave handbook of childhood studies. eds. Qvortrup J. Corsaro W. A. Honig M. S. (London/New York: Palgrave), 159–174.

[ref3] Bond Stockton K. (2009). The queer child, or growing sideways in twentieth century. Durham: Duke University Press.

[ref4] Brinch R. (2018). *Att växa sidledes. Tematik, barnsyn och konstnärlig gestaltning i Suzanne Ostens scenkonst för unga*. [dissertation]. Stockholm: Stockholm University.

[ref5] Cvetkovich A. (2003). An archive of feelings. Trauma, sexuality and lesbian public cultures. Durham/London: Duke University Press.

[ref6] Elnan M. (2009). Staging the impossible for young audiences. Preliminary findings in a research project. Youth Theatre J. 23, 39–47. doi: 10.1080/08929090902851569

[ref7] Gjervan E.-K. (2013). Lekens dramaturgi – nye muligheter for barneteatret? Nord. Early Child. Educ. Res. J. 6, 1–11. doi: 10.7577/nbf.385

[ref8] Göteborgs Stadsmuseum. (2016). RÖST-prov med Suzanne Osten på Göteborgs stadsmuseeum, Available online at:https://www.mynewsdesk.com/se/goeteborgs-stadsmuseum/pressreleases/roest-prov-med-suzanne-osten-1342373. Accessed 15 May 2025.

[ref9] Helander K. (1999). Från sagospel till barntragedi. Pedagogik, förströelse och konst i 1900-talets svenska barnteater. Stockholm: Carlssons.

[ref10] Helander K. (2011). Den var rolig och så lärde man sig någonting, men jag kommer inte på vilket det var. Om barnteater och receptionsforskning. Locus 23, 81–97.

[ref11] Helander K. (2018). “Den barnförbjudna barnkulturen” in Barnkulturens gränsland. eds. Wester M. Öhrn M. (Stockholm: Centrum för barnkulturforskning nr 51), 20–36.

[ref12] Helander K. (2024). “The movie ended quite well, but not that well, and I liked that because that ´s how it is in reality” in Reception of and debates on Suzanne Osten ´s films for children, Swedish Children ´s cinema. History, ideology and aesthetics. ed. Janson M. (London: Palgrave Macmillan), 159–174.

[ref13] Helander K. Osten S. (2007). Konstnärlig barnteater - en dialog om betydelsen av scenkonst för barn, paper från konferensen “Börn & kultur i det 21.Århundrede – Hvad ved vi?”. BIN – Norden: Barnekulturforskning i Norden, 1–15.

[ref14] Helander K. Osten S. (2010). “Barnets familjer på teaterscenen, Suzanne Osten och Karin Helander i samtal” in Barnets familjer ur barnkulturella perspektiv. eds. Banér A., P. Ulf (Stockholm: Centrum för barnkulturforskning nr 43), 45–53.

[ref15] Helander K. Zern L. (2010). Det skall åska och blixtra kring vår teater Stockholms stadsteater 1960–2010. Stockholm: Bonniers.

[ref16] Johansson B. (2006). Befrielsen är nära: Feminism och teaterpraktik i Margareta Garpes och Suzanne Ostens 1970-talsteater. Stehag: Symposion: Gothenburg University.

[ref17] Lee N. (2001). Childhood and society. Growing up in an age of uncertainty. London: Open Press University.

[ref18] Lidén E. (2022). *Barnteater som händelse. Barnpublik och vuxenvärldar vid Kulturhuset Stadsteaterns scen i Skärholmen*. [dissertation]. Stockholm: Stockholm University.

[ref19] Lorentzon Y. Brinch R. Lund A. (n.d.). The (im)possibility of children’s participation in professional theatre staging migration. Eur. J. Polit. Cult. Sociol. [Epub ahead of print].

[ref20] Osten S. (1998). “Psyksjuka slängs ut”. Min Schizofrena mamma slapp åtminstone Bo på gatan, skriver Suzanne Osten. Dagens Nyh.

[ref21] Osten S. (2006). *Mina meningar: Essäer, artiklar, analyser 1969–200*2. Möklinta: Gidlunds förlag.

[ref22] Osten S. (2009). Nio pjäser på Unga Klara. Stockholm: Themis förlag.

[ref23] Osten S. (2013). Det allra viktigaste. Dagbok. Möklinta: Gidlunds förlag.

[ref24] Osten S. (2015). “Är teatern farligare än verkligheten?”, Dagens Nyheter, Available online at:https://www.dn.se/kultur-noje/kulturdebatt/suzanne-osten-ar-teatern-farligare-an-verkligheten/. [Accessed 18 March 2025].

[ref25] Osten S. (2021). Vem tror hon att hon är, Suzanne Osten? En självbiografi i tre akter. Stockholm: Ordfront förlag.

[ref26] Osten S. Högberg A. (1998). Flickan, mamman och soporna. Stockholm: Bromberg.

[ref27] Osten S. Uddenberg E. Barany A. (2014). Unga Klara presenterar Lammungarnas fest av Leonora Carrington regi Suzanne Osten. Stockholm: Unga Klara.

[ref28] Rosenberg T. (2004). Besvärliga Människor. Teatersamtal med Suzanne Osten. Stockholm: Atlas.

[ref29] Rosenberg T. (2009). On feminist activist aesthetics. J. Aesthet. Cult. 1, 1–12. doi: 10.3402/jac.v1i0.4619, PMID: 30636950

[ref30] Rosenberg T. (2012). Ilska, hopp och solidaritet. Med feministisk scenkonst in i framtiden. Stockholm: Atlas.

[ref31] Skjerfving A. (2015). Barn till föräldrar med psykisk ohälsa. Barndom och uppväxtvillkor. [Stockholm University]: Stockholm.

[ref32] SOU (1997). Röster om barns och ungdomars psykiska hälsa. Stockholm: tatens offentliga utredningar.

[ref33] SOU (1998). Det gäller livet. Stöd och vård till barn och ungdomar med psykiska problem. Stockholm: Statens offentliga utredningar.

[ref34] Sparby M. (Ed.) (1986). Unga Klara. Barnteater som konst. Stockholm: Gidlund.

[ref35] Švachová R. (2016). The childish unga klara. Contemporary Swedish children’s theatre and its experimental aesthetics. Brunner Beiträge zu Germanistik und Nordistik 30, 51–63. doi: 10.5817/BBGN2016-1-5

[ref36] Svens C. (2002). Regi med feministiska förtecken. Suzanne Osten på teatern. [Umeå University]: Hedemora: Gidlunds.

[ref37] Svensk Filmdatabas (2025). Available online at:https://www.svenskfilmdatabas.se/sv/item/?type=film&itemid=5933#about-the-film. [Accessed 18 March 2025].

[ref38] Svenska Dagbladet, “Estetiska ämnen inte obligatoriskt för elever”, (2018) [Accessed 17 May 2025]. Available online at:https://www.svd.se/a/BJqvLE/estetiska-amnen-inte-obligatoriskt-for-elever

[ref39] Van de Water M. (2012). *Theatre*, youth, and culture. A critical and historical exploration. London/New York: Palgrave.

[ref40] von Zweigberg H. (1990). Barndom, feminism, galenskap: Osten om Osten. Stockholm: Alfabeta.

[ref41] Winnicott D. (1995). Lek och verklighet. Stockholm: Natur & Kultur.

[ref42] Young-Bruehl E. (2013). Childism. Confronting prejudice against children. Yale: Yale University Press.

